# Ego-Resiliency and Coping Styles in Patients with Generalized Anxiety Disorder During the COVID-19 Pandemic

**DOI:** 10.3390/medicina61122234

**Published:** 2025-12-17

**Authors:** Karina Badura-Brzoza, Patryk Główczyński, Paweł Dębski, Michał Błachut, Sebastian Binyamin Skalski-Bednarz

**Affiliations:** 1 Clinical Department of Psychiatry, Faculty of Medical Sciences in Zabrze, Medical University of Silesia in Katowice, 40-055 Katowice, Poland; kbbrzoza@sum.edu.pl (K.B.-B.); pdebski@sum.edu.pl (P.D.); mblachut@sum.edu.pl (M.B.); 2 Institute of Psychology, Humanitas Academy in Sosnowiec, 41-200 Sosnowiec, Poland; 3 Faculty of Philosophy and Education, Catholic University of Eichstätt-Ingolstadt, 85072 Eichstätt, Germany; sebastian.skalski@ku.de

**Keywords:** ego-resiliency, coping, anxiety, GAD

## Abstract

*Background and Objectives*: In the difficult socio-economic situation of recent years, particular attention has been paid to factors that may have a protective effect on mental health. One of these factors is the individual resource known as ego-resiliency. The aim of the study was to assess ego-resiliency as a potential protective factor against stress symptoms and to determine effective coping strategies in a group of patients with Generalized Anxiety Disorder (GAD) during the COVID-19 pandemic. *Materials and Methods*: The study was conducted from March 2020 to March 2022 among patients attending a Mental Health Clinic in the Upper Silesian metropolitan area of Poland. A total of 68 participants with a confirmed diagnosis of GAD (42 women, 26 men) completed the Ego-Resiliency Scale (ER89-R12), the Coping Inventory for Stressful Situations (CISS), and the Perceived Stress Scale (PSS-10). *Results*: The mean score on the ER89-R12 was 32.85 ± 6.52 points, with no significant gender differences. The mean PSS-10 score was 22.48 ± 5.61, corresponding to a moderate to high perceived stress level. Significant negative correlations were found between ego-resiliency and perceived stress, and positive correlations between ego-resiliency and task-focused coping. *Conclusions*: Ego-resiliency may serve as a protective factor against stress and promote adaptive, task-focused coping strategies in patients with Generalized Anxiety Disorder. Strengthening ego-resiliency may be a relevant therapeutic target in clinical interventions.

## 1. Introduction

In recent decades, the perception of mental health has shifted substantially, with increasing recognition that it is as important as physical health [1]. Major global crises such as the COVID-19 pandemic have highlighted the psychological consequences of collective stress, leading to widespread emotional and social distress alongside physical health challenges. These circumstances have intensified the prevalence of mental health problems, both among individuals with pre-existing psychiatric conditions and those who developed anxiety or depressive symptoms in response to stressors that exceeded their coping capacities [2,3]. In this context, research has increasingly focused on identifying psychological factors that may protect mental well-being under adverse conditions. One such factor is ego-resiliency, which refers to an individual’s capacity to adapt flexibly and resourcefully to changing or stressful circumstances [4,5,6]. It represents a dynamic personality trait that enables individuals to perceive stressors as challenges, regulate emotions effectively, and mobilize both internal and external coping resources [7,8,9]. Ego-resiliency is conceptually related to the broader construct of resilience, but it emphasizes flexibility in self-regulation and adaptation to environmental demands [10]. According to Block and Kremen [11], ego-resiliency constitutes a relatively stable disposition that facilitates adjustment to constantly changing life conditions and supports persistence, emotional balance, and constructive responses to adversity. Empirical studies have shown that individuals with higher ego-resiliency tend to experience fewer stress-related symptoms and demonstrate greater psychological flexibility in coping with adversity [12,13,14]. However, most of these findings come from community or mixed samples, while evidence from clinical populations—particularly individuals diagnosed with Generalized Anxiety Disorder (GAD)—remains limited [15,16,17]. GAD is characterized by chronic, excessive anxiety and difficulty controlling worry, often accompanied by physiological arousal and somatic tension. Prolonged exposure to uncertainty and stress, such as that experienced during the COVID-19 pandemic, may exacerbate these symptoms. Understanding how ego-resiliency interacts with coping mechanisms in this population could therefore inform both therapeutic strategies and preventive interventions. Coping styles, as described by Endler and Parker [18], represent habitual patterns of managing stress and include task-oriented, emotion-oriented, and avoidance-oriented coping. Ego-resiliency may influence both the selection and the effectiveness of these coping strategies, promoting adaptive behaviors and reducing maladaptive responses. Accordingly, the present study aimed to examine the relationship between ego-resiliency, perceived stress, and coping styles in patients with GAD during the COVID-19 pandemic. It was hypothesized that higher ego-resiliency would be associated with lower levels of perceived stress and greater engagement in adaptive, task-focused coping strategies.

## 2. Materials and Methods

The study was conducted between March 2020 and March 2022. Participants were recruited from the Mental Health Clinic (PZP) located in the Upper Silesian Metropolis, Poland. Initially, 150 patients with at least one year of medical history at the clinic were invited to participate. A total of 89 completed questionnaires were returned and included in the final analysis.

Participants met the diagnostic criteria for Generalized Anxiety Disorder (GAD) according to the DSM-5. Exclusion criteria included the presence of severe psychiatric disorders (e.g., psychosis, bipolar disorder), acute somatic illness, or inability to complete the questionnaires. All participants provided written informed consent.

The following psychometric tools and questionnaires were used to assess the studied parameters:

Original demographic data questionnaire. This questionnaire was constructed to control for sociodemographic variables.

The Ego-Resiliency Scale (ER89-R12) is a questionnaire used to measure mental resilience, in a Polish adaptation. The questionnaire consists of 12 questions rated on a four-point scale. The overall result is obtained by summing up the partial results from statements. The tool also allows for assessment in two subscales: optimal regulation and openness to life experience. The reliability of the tool was assessed using the Cronbach’s α coefficient and amounted to 0.82 [7].

CISS—Coping Inventory for Stressful Situations. The CISS questionnaire is used to measure the intensity of behavioral tendencies that people engage in stressful situations. The tool allows you to identify three styles of behavior towards stressful situations: the style focused on the task, the style focused on emotions and the style focused on avoidance. It is also possible to assess two forms of avoidance-focused style-engaging in substitute activities and seeking social contacts. CISS consists of 48 statements regarding various behaviors to which the respondents refers on a five-point scale determining their frequency, where one means “never” and five means “very often”. To obtain results, the scores marked by the examinee for the items included in the individual scales are summed up. The results can be normalized using the sten scale. The internal consistency index for individual scales ranged from 0.78 to 0.90 [19].

Perceived Stress Scale (PSS-10). This is a questionnaire used to examine the intensity of stress related to situation. This rating covers four past weeks. Stress is described in that tool as a reaction to intractable experiences. The scale consists of ten items to which the respondent refers on a five-point scale. In that terms, zero means “never” and four means “very often”, and score ranges from zero to forty. The higher the score, the greater the perceiving stress. The test results are normalized so that they can be presented using the sten scale. The Cronbach’s α coefficient for the entire tool was 0.86 [20].

### 2.1. Statistical Analysis

Standard statistical procedures were used in the analyses. Normality of distributions was assessed based on the results of the Shapiro–Wilk test. Due to the lack of normal distributions, non-parametric procedures were used for most variables. The Mann–Whitney U test was used to assess the significance of differences between the study groups. To assess the relationships between the data, Spearman’s rank correlation coefficient was used. The significance level of α ≤ 0.05 was assumed as statistically significant. Calculations were performed in Statistica version 13.3 and Excel 2016.

### 2.2. Ethical Consideration

The Bioethics Committee of the Medical University of Silesia in Katowice agreed to conduct the research (PCN/0022/KBI/67/21). The patients provided their written informed consent to participate in this study.

## 3. Results

The study included 68 participants: 42 women (mean age 43.06 ± 14.89 years) and 26 men (mean age 40.84 ± 16.72 years). All participants were diagnosed with GAD. Sociodemographic characteristics are presented in Table 1.

### 3.1. Ego-Resiliency

The mean ego-resiliency score was 32.85 ± 6.52. Women scored 33.29 ± 6.19, and men scored 32.28 ± 5.67; the difference was not statistically significant (Table 1 and Table 2).

### 3.2. Perceived Stress

The mean PSS-10 score was 22.48 ± 5.61, corresponding to a high level of perceived stress. Women scored 23.14 ± 5.91, and men 21.36 ± 5.16; the difference was not statistically significant (Table 2 and Table 3).

### 3.3. Coping Styles

The mean task-oriented coping score was 51.70 ± 9.05. Women scored 50.65 ± 9.22 and men 53.12 ± 8.93; these values represent a low-to-average level and did not differ significantly between genders.

The mean emotion-oriented coping score was 46.79 ± 9.01 (45.50 ± 9.26 for women, 48.76 ± 8.48 for men), corresponding to an average level for both genders.

The mean avoidance-oriented coping score was 44.45 ± 6.95 (44.45 ± 7.84 for women, 44.32 ± 5.41 for men), also at an average level (Table 2 and Table 3).

### 3.4. Correlational Analysis

Across the entire sample, ego-resiliency was significantly and negatively correlated with perceived stress (r = −0.414), indicating that higher ego-resiliency was associated with lower perceived stress. Additionally, a positive, nearly moderate correlation was found between ego-resiliency and task-oriented coping (r = 0.287) (Table 4).

## 4. Discussion

Coping styles represent consistent patterns of strategies that individuals employ to manage stressful or challenging situations. The effectiveness of these strategies depends on both individual personality traits and external situational factors, including the nature of the stressor, cognitive appraisal, and availability of social support [21]. Among personality-related factors, ego-resiliency has been identified as one of the key determinants of adaptive coping [10].

Ego-resiliency reflects an individual’s ability to flexibly regulate behavior and emotions in response to environmental demands. Individuals with higher ego-resiliency typically appraise stressors as challenges rather than threats, maintain cognitive control, and employ constructive coping strategies [22,23]. They tend to experience fewer negative emotions and demonstrate greater tolerance for uncertainty—an especially important characteristic in patients with Generalized Anxiety Disorder (GAD), who often struggle with persistent and uncontrollable worry.

In the present study, higher ego-resiliency was associated with lower perceived stress and greater reliance on task-focused coping, whereas lower ego-resiliency corresponded to greater emotional distress. These findings are consistent with previous research highlighting the protective role of resilience-related traits in anxiety and stress regulation [10,22,24,25,26]. During the COVID-19 pandemic, psychological flexibility and resilience were shown to mitigate the adverse effects of prolonged uncertainty and social isolation, supporting adaptive functioning even among clinical populations [23,27].

Our results also expand the understanding of coping mechanisms in GAD. The predominance of emotion-focused and avoidance coping styles observed in this group aligns with earlier reports indicating that individuals with anxiety disorders often rely on emotion regulation strategies aimed at alleviating tension rather than addressing stressors directly [27,28]. Although such strategies may provide temporary relief, they can reinforce maladaptive cognitive patterns, perpetuate anxiety, and diminish perceived self-efficacy.

In contrast, task-focused coping is generally considered adaptive, as it involves problem-solving, cognitive restructuring, and active engagement in stress management. The positive correlation between ego-resiliency and task-focused coping observed in this study suggests that enhancing ego-resiliency could promote more constructive coping strategies and improve emotional regulation in patients with GAD.

Furthermore, these findings underscore the importance of incorporating resilience-building interventions into therapeutic programs for anxiety disorders. Psychological techniques such as cognitive-behavioral therapy (CBT), mindfulness-based interventions, and emotion regulation training have been shown to strengthen resilience and coping flexibility [28]. These interventions may help patients shift from avoidance or emotion-based responses toward more problem-oriented strategies, thereby fostering a greater sense of mastery and control.

Although this study was conducted during the unique socio-historical context of the COVID-19 pandemic, the results may also reflect broader patterns of stress reactivity and coping in modern society—one characterized by chronic exposure to global and regional crises. Such contextual factors likely contribute to elevated stress levels in psychiatric populations, including patients with GAD.

Finally, while this study’s cross-sectional design does not allow for causal inference, the observed associations suggest that ego-resiliency may act as a buffer between perceived stress and coping style, reducing vulnerability to maladaptive responses under chronic stress conditions. Future longitudinal research is warranted to confirm this potential moderating role and to determine whether ego-resiliency can be effectively enhanced through targeted psychotherapeutic interventions.

## 5. Conclusions

The present study demonstrates that ego-resiliency is associated with lower perceived stress and greater engagement in adaptive, task-focused coping strategies among patients with Generalized Anxiety Disorder (GAD). These findings suggest that ego-resiliency functions as a protective psychological resource, supporting flexible adaptation under prolonged stress conditions such as those experienced during the COVID-19 pandemic.

From a clinical perspective, the results highlight the relevance of integrating resilience-enhancing interventions—including cognitive-behavioral, mindfulness-based, and emotion regulation approaches—into treatment programs for anxiety disorders. By fostering ego-resiliency, clinicians may help patients shift from maladaptive emotion- or avoidance-based strategies toward more effective coping mechanisms that promote long-term psychological stability.

Although causality cannot be inferred from this cross-sectional design, the study provides valuable insights into the interplay between resilience and coping in a vulnerable clinical population. These findings point to promising directions for therapeutic innovation and preventive care.

### Limitations

Several Limitations Should Be Acknowledged:

- First, the study employed a cross-sectional design and relied on self-report measures, which may introduce response bias and preclude causal interpretations.
- Second, the absence of a control group limits the ability to compare ego-resiliency levels between clinical and non-clinical populations.
- Third, important contextual variables such as pandemic-related stressors, treatment delays, and socio-economic burden were not directly assessed and therefore should be treated as background rather than measured constructs.
- Fourth, the relatively small sample size (n = 68) and its subdivision by gender reduce statistical power and limit generalizability.
- Finally, data regarding education, marital status, comorbidities, and medication use should be reported in future studies to improve transparency and replicability.

Despite these limitations, the study provides novel evidence supporting the potential role of ego-resiliency as a protective factor in patients with GAD. Future research using longitudinal and intervention designs is warranted to verify whether enhancing ego-resiliency can reduce perceived stress and promote adaptive coping over time.

## Figures and Tables

**Table 1:** Sociodemographic characteristics of the sample.

Variable	n/M (SD)	%
Age (years)	42.90 (SD = 15.52)	
Gender: Female	42	63
Gender: Male	26	37
Cohabitation: Yes	47	69.1
Marital status: In a relationship/Married	42	61.8
Education: Vocational	12	17.6
Education: Secondary	35	51.5
Education: Higher	21	30.9
Duration of illness (years)	5.71 (SD = 4.75)	

**Table 2:** Descriptive statistics of variables for the entire study group (n = 68).

n = 68	Mean	St. Dev.	Median	Min.	Max.
**ER**	35.854	5.689	36.000	32.000	40.000
**PSS-10**	22.478	5.617	22.000	30.000	40.000
**CISS-SSZ**	51.706	9.046	52.000	37.000	74.000
**CISS-SSE**	46.794	9.012	47.000	27.000	70.000
**CISS-SSU**	44.456	6.955	45.000	30.000	62.000

ER—Ego-resiliency scale; PSS-10—Perceived Stress Scale; CISS-SSZ—Task-based model of coping with stressful situations; CISS-SSE—Model of Coping with Stressful Situations based on emotions; CISS-SSU—Model of Coping with Stressful Situations based on avoidance.

**Table 3:** Difference between genders.

	Women (n = 42)			Men (n = 26)			Mann–Whitney U	
	Median	Q1	Q3	Median	Q1	Q3	Z	p
**ER**	33	29	37	33	26	37	0.248	0.804
**PSS-10**	24	20	27	21	19	24	1.561	0.118
**CISS-SSZ**	49	46	54	52	47	56	−1.175	0.240
**CISS-SSE**	47	41	50	46	44	52	−0.747	0.455
**CISS-SSU**	45	40	50	44	41	47	0.026	0.979

ER—Ego-resiliency scale; PSS-10—Perceived Stress Scale; CISS-SSZ—Task-based model of coping with stressful situations; CISS-SSE—Model of Coping with Stressful Situations based on emotions; CISS-SSU—Model of Coping with Stressful Situations based on avoidance; Q1—lower quartile, Q3—upper quartile; Z—Mann–Whitney U statistic result; p—probability for the Mann–Whitney U test.

**Table 4:** Relationships between ego-resiliency, perceived stress and styles of coping with stress (n = 68).

n = 68	ER	PSS-10	CISS-SSZ	CISS-SSE	CISS-SSU
**ER**	1.000	−0.414 *	0.287 *	−0.228	0.064
**PSS-10**		1.000	−0.457 *	0.076	−0.180

ER—Ego-resiliency scale; PSS-10—Perceived Stress Scale; CISS-SSZ—Task-based model of coping with stressful situations; CISS-SSE—Model of Coping with Stressful Situations based on emotions; CISS-SSU—Model of Coping with Stressful Situations based on avoidance. * *p* < 0.01.

## Data Availability

Raw data were generated at Medical University of Silesia. Derived data supporting the findings of this study are available from the corresponding author P.G on request.
